# Frequency TMJ disc displacements

**DOI:** 10.4317/jced.63252

**Published:** 2025-10-17

**Authors:** Nataliia O. Savychuk, Vasil V. Pekhnyo, Roman G. Osnach

**Affiliations:** 1 Vice-Rector for Science (Department of Science) Employment Shupyk National Healthcare University of Ukraine, Kyiv; 2 Associate Professor, department of orthopedic dentistry, digital technologies and implantology, Shupyk National Healthcare University of Ukraine, Kyiv

## Abstract

**Background:**

Temporomandibular joint (TMJ) disorders are characterized by alterations in joint structure and function, with disc displacement being one of the most prevalent pathological findings. The frequency, typology, and clinical correlates of these displacements, particularly with regard to intra-articular effusion, remain insufficiently explored.

**Material and Methods:**

The retrospective study included 244 MRI examinations of patients with TMJ disorders. Imaging was performed using standardized protocols on 1.5 T MRI scanners. Disc position was classified according to established radiological criteria. The prevalence and distribution of various disc displacement types, as well as the presence and degree of joint effusion, were systematically evaluated

**Results:**

Anterior disc displacement was identified in 9.84% (right) and 8.61% (left) TMJ of unilateral cases, and in 17.21% (right) and 16.80% (left) TMJ of bilateral cases. Partial anterior disc displacement was observed in 8.61-10.66% of unilateral cases and 15.16-15.57% of bilateral cases. Rotational displacements were found in 6.15-6.56% of unilateral cases and 12.70-13.11% of bilateral cases, while lateral/medial and posterior displacements ranged from 0.41% to 3.69%. A normal disc position was present in 20.90-22.95% of joints. Joint effusion was most often recorded in cases of anterior and partial anterior displacements, predominantly minimal (2.9-5.3%) or small (1.2-2.9%) in degree. In most cases of lateral, posterior, and rotational displacement, as well as with normal disc position, effusion was absent.

**Conclusions:**

Anterior disc displacement is the most prevalent TMJ pathology among symptomatic patients, with a frequency up to 17.2% in bilateral cases. Effusion is most closely associated with anterior and partial anterior displacements but is typically minimal or absent. These findings support a predominantly mechanical, rather than inflammatory, etiology for disc displacement in this patient population.

## Introduction

Temporomandibular disorders (TMDs), encompass a range of conditions that affect the temporomandibular joint (TMJ), masticatory function, and muscle integrity, and can lead to headaches and issues with related structures (1). The global prevalence of TMD with symptoms is 41%, while the rate is 56% for those with at least 1 clinical sign. The prevalence of TMD-related pain is 23.4% among children and 36.9% among adults (2 , 3). For the past 5 years, numerous studies have appeared assessing the prevalence of signs of TMD in the adult population using magnetic resonance imaging (MRI) for diagnosis. Population-wide data indicate that 50-75% of people report at least one sign of TMD during their lifetime, while 20-25% report at least one symptom of TMD (3). At the same time, clinically significant TMDs (accompanied by pain or severe dysfunction) occur in approximately 5-12% of adults (4). According to the latest global estimates, TMD affects about 30-34% of the world's population, with women getting sick almost twice as often as men (5 , 6). The higher prevalence in the female population was thought to be the consequence of different pain tolerance and weaker joint structure, as well as hormonal influences (7).Females are almost twice as prone to TMD, and the peak occurrence of these disorders is between 20 and 40 years of age, with pain in the TMJ as the most common symptom (4 , 8). In the adult population, its prevalence, according to various sources, ranges from 20% to 30%. Thus, in the mentioned German study, 25% of the examined had joint noises (clicking or crunching) during jaw movements. Other studies confirm a similar level (about 15-25%, depending on age and gender). Crepitus (friction or "crunching" in the joint) occurs less often than a soft click and is usually associated with degenerative changes in the joint - it is detected mainly in older people or with osteoarthritis of the TMJ. Subjectively, patients do not always notice murmurs: only 9-13% of adults themselves report clicking or crunching in the jaw (9). These results confirm of MRI studies: even asymptomatic people often have a disc displacement that causes clicking, but they do not regard it as a problem. According to a modern review, approximately 30% of people without symptoms have MRI signs of disc displacement or other intra-articular changes (10). On the other hand, among patients with TMD, the proportion of people with joint murmurs is much higher - up to 50-60% (11). MRI is now recognized as the "gold standard" of imaging for assessing the position and condition of the articular disc and bone structures of the TMJ (12). According to MRI, intra-articular disorders (disc displacements) are the most common category of TMD. Disc displacement with reduction is diagnosed in from 10 to15% of the general population (often asymptomatic) and accounts for more than half of all TMD cases. Disc displacement without reduction is less common (1-3% of the population), but among patients with TMD, the proportion of DDwoR reaches 5-15%. MRI confirms that an unrepositioned disc is often combined with arthrosis: up to 66% of such cases have further complications in the form of osteoarthritis (13). Tasaki et al. (1996), in his study emphasize the most common types of temporomandibular joint (TMJ) disk displacement in patients with clinical signs of dysfunction are anterolateral disk displacement (23.3%) and anterior disk displacement (22.6%), while a normal superior disk position was found in only 18.1% of patients; other types of displacement, such as partial anterior disk displacement in the lateral part of the joint (8.2%), partial anterior disk displacement in the medial part of the joint (0.8%), rotational anteromedial disk displacement (4.3%), lateral disk displacement (4.5%), medial disk displacement (4.1%), and posterior disk displacement (0.6%), were observed less frequently. In the control group of symptom-free volunteers, a bilateral superior disk position was found in 70.2%, and various forms of disk displacement in 29.8% of joints, mainly as anterolateral disk displacement (8.8%) and partial anterior disk displacement in the medial part of the joint (6.1%). Thus, disk displacement is significantly more common in patients with clinical symptoms, but nearly one third of asymptomatic individuals also had disk displacement on MRI, confirming its potential for asymptomatic presentation (14).

## Material and Methods

This retrospective study included 244 consecutive MRI examinations of adult patients presenting with at least one clinical symptom of temporomandibular joint (TMJ) dysfunction-such as joint sounds, pain, or restricted jaw movement - who were referred for imaging prior to interdisciplinary treatment. Inclusion and Exclusion Criteria: Inclusion criteria were: adult patients presenting with at least one clinical symptom suggestive of TMJ dysfunction (such as joint sounds, pain, or limited jaw movement) and referred for MRI evaluation prior to interdisciplinary treatment. Exclusion criteria were: history of previous TMJ surgery, recent maxillofacial trauma, congenital craniofacial anomalies, inflammatory or systemic joint diseases (such as rheumatoid arthritis), or incomplete MRI data. MRI Scans were performed on Siemens MAGNETOM Aera (1.5 T) and Philips ACHIEVA (1.5 T) scaners The imaging protocol included T1, T2 and PD-weighted sequences in oblique and oblique coronary planes, with slice thickness of 2 mm. These projections were chosen by us for their improved bisualisation of disk position and joint structures compared to standart sagittal and coronal planes, The study were approved by the protocol of the Commission on Ethics and Academic Integrity of the Shupyk National University of Health of Ukraine. We chose the classification according to the criteria of TMJ disc displacements by Tasaki et al. (1996)(14), which provide a comprehensive categorization of TMJ disc displacement types. Based on MRI findings, patients were divided into two groups: Group 1 (unilateral TMJ involvement) and Group 2 (bilateral TMJ involvement). For frequency analysis, the following displacement types were assessed: anterior disc displacement, partial anterior disc displacement (lateral/medial), rotational anterolateral/anteromedial, lateral/medial disc displacement, posterior disc displacement, and normal disc position (no displacement).

## Results

Analysis of 244 TMJ MRI examanations divided into unilateral (Group 1) and bilateral (Group 2) dislocation groups revealed that anterior disk displacement was the most frequent finding. In the unilateral cases, anterior disk displacement was indetified in 9.84% of cases on the right and 8.61% on the left TMJs , in bilateral cases, the prevalence increased up to 17.21%(right TMJ) and 16.80%(left TMJ). Partial anterior displacement disc was also common, accounting for 8.61-10.66% in unilateral and 15.16-15.57% in bilateral study groups. Other of displacement types (rotational, lateral/medial, and posterior) were lessfrequent , ranging from 0.41% to 13.11%. Effusion was most frequently associated with anterior and partial anterior disc displacements In Group 1, the highest frequency of effusion was observed with anterior displacement of the disc of the right joint (minimal effusion in 7 cases - 2.9%, small - in 4 cases - 1.6%, moderate - in 1 case - 0.4%), as well as with partial anterior displacement of the left joint (minimal effusion in 7 cases - 2.9%, small - in 4 cases - 1.6%, moderate - in 1 case - 0.4%). With rotational displacements, effusion was detected less frequently: for the right TMJ - minimal in 4 cases (1.6%), small in 2 cases (0.8%), for the left - minimal in 4 cases (1.6%), small in 2 cases (0.8%), moderate in 2 cases (0.8%). For lateral/medial displacements, posterior displacement and with normal disc position, in most cases, the effusion was absent or occurred sporadically. No pronounced and increased forms of effusion were recorded in this group. In Group 2, effusion was most often recorded with anterior displacement of the disc in both the right and left joints: minimal effusion was recorded in 13 cases for each side (5.3%), small in 6-7 cases (2.5-2.9%), moderate in 3 cases (1.2%), and increased effusion in 1 case for each side (0.4%). A similar trend can be observed for partial anterior displacements: minimal effusion in the right joint - in 12 cases (4.9%), in the left - also in 12 (4.9%); small - 7 cases (2.9%) on each side; moderate and increased - isolated cases (0.8%). In the case of rotational, lateral/medial and posterior displacements, as well as in normal disc position, effusion was absent in most cases. Thus, regardless of the side of the lesion, the highest frequency of effusion is characteristic of anterior and partial anterior disc dislocations, while in other types of dislocations or with a normal disc position, effusion was practically not recorded. Separately, it is worth emphasizing that the absence of effusion in the joint in the presence of disc dislocation was quite common. In particular, in Group 1, the absence of effusion was most often recorded in lateral and posterior dislocations, as well as in partial anterior and rotational displacements: for example, in partial anterior disk displacement, effusion was absent in 11 cases on the right (4.5%) and 14 cases on the left (5.7%), with rotational displacement - in 8 cases on each side (3.3%). In Group 2, a similar picture was observed: the absence of effusion was observed in 15 cases on the right (6.1%) and 16 cases on the left (6.6%), with rotational displacement - in 13 cases on each side (5.3%), and with lateral and posterior dislocations - in 5 cases on the right (2.0%) and 4-5 cases on the left (1.6-2.0%). Therefore, even in the presence of disc displacement, most of the cases have no signs of effusion, which further emphasizes the predominantly mechanical predictor of abnormalities in the selected cohort of patients (Table 1).

Table 1

## Discussion

An analysis of 244 MRI examinations of the temporomandibular joint (TMJ) in patients with TMD showed that most patients had an anterior disc dislocation in the position of habitual occlusion, which consistent with the literature, according to which disc dislocation with preserved reduction is one of the most common intra-articular pathologies of TMJ and accounts for about 41% of TMD diagnoses. The lateral and posterior displacements of the disc were significantly rarer, which is consistent with current ideas about the predominantly anterior nature of the displacement (15). Joint effusion (accumulation of synovial fluid) on MRI was detected in more than half of the cases, which is comparable to the reports from other researchers (e.g., ~46.5% of TMD patients according to MRI data(16) Disc dislocations and effusion were somewhat more common in the right TMJ, although bilateral lesions were also common. Factors contributing to disc displacement include: chronic microtraumatization or acute trauma, structural changes in joint components, degenerative lesions of the articular surfaces, occlusive anomalies, hyperactivity of the lateral cr/silt muscle, hypermobility (against the background of connective tissue dysplasia, or acquired as a result of existing orthodontic pathology during the period of milk and malocclusion), as well as weakness or stretching of the ligamentous apparatus of the joint (3 , 15 , 17). These structural changes are predictors that can begin the pathogenesis of TMD development, one of the manifestations of which is a violation of the physiological position of the TMJ disc. Joint effusion in the TMJ is usually considered as a marker of the inflammatory process in the joint. On MRI, the effusion is manifested by a zone of hyperintense signal on T2-weighted images, which corresponds to the accumulation of synovial fluid due to inflammation of the synovial membrane (18). The mechanism of its development is associated with synovitis: the dislocated disc injures the articular structures, causing an inflammatory reaction and increased production of joint fluid. This explains why effusion is often combined with disc dislocation. Indeed, in more severe cases, such as anterior disc dislocation without reduction, there is a significant accumulation of fluid in the joint (19). At the same time, the presence of effusion is not always accompanied by clinical symptoms of inflammation or pain. Some modern studies have shown that there is no significant relationship between the presence of effusion and pain syndrome in the TMJ (20). It is believed that effusion can manifest itself even in the absence of signs of active inflammation or arthralgia (18). The sex factor can influence the tendency to effusion: according to the latest data, women with TMD have a significantly higher risk of developing joint effusion (about 5 times higher than in men) (20), which probably reflects hormonal or anatomical factors in the pathogenesis of this condition. Comparing our results with current research demonstrates both consistency and some differences. The dominance of anterior disc dislocations that we have established is consistent with most publications: anterior disc displacement is known to be the most common type of intra-articular TMJ disorder (17). Also, a large proportion of cases of dislocation with reduction in our sample coincides with the data of clinical observations of patients with TMD (15). The incidence of articular effusion in our study is within a range similar to other MRI tests: for example, according to Lee et al. (2024), effusion is found in ~46.5% of patients with TMD (16). While Mizuhashi et al. Effusion was reported in 70 of the 97 examined joints (~72%) with disc dislocations Such a difference in frequency may be due to temporal differences in the requests of the examinees, age and racial differences (19). Thus, the literature confirms the high prevalence of effusion in internal TMJ disorders, especially in cases of non-repositional displacement of the disc. Our results also showed a tendency towards a bilateral nature of the lesion: many patients had disc dislocations and effusion in both joints. This is in line with generally accepted statistics found in similar publications. On the other hand, individual studies record the asymmetry of the lesion: for example, in patients with pronounced facial asymmetry, anterior disc dislocations and bone changes occur more often on the left side, which is a promising direction for subsequent studies (21). In general, the results obtained by us are in good agreement with the current literature data on the pathogenesis and prevalence of intra-articular changes in TMD, confirming that anterior disc dislocation is a leading pathology, often accompanied by effusion, and their manifestation may depend on the severity of the condition and the characteristics of the patient. Limitations This study has several limitations. First, its retrospective design may introduce selection bias despite the use of clear inclusion and exclusion criteria. Second, all data were obtained from a single clinical center, which may limit the generalizability of the findings to broader populations. Third, the analysis was based exclusively on MRI results without systematic correlation to clinical signs and symptoms, precluding direct assessment of the clinical significance of the imaging findings. Finally, the lack of longitudinal follow-up did not allow evaluation of the progression or outcomes associated with specific types of disc displacement and joint effusion.

## Conclusions

1. The most common type of TMJ disc dislocation in both groups was anterior dislocation, which was found in 9.84% of cases in the right and 8.61% in the left joint among patients with unilateral lesions, as well as in 17.21% and 16.80%, respectively, in bilateral lesions. 2. Partial anterior disc dislocation (lateral/medial) ranked second in frequency, accounting for 8.61-10.66% in the group of unilateral lesions and 15.16-15.57% in the group of bilateral lesions. 3. Other types of dislocations (rotational, lateral/medial, posterior) were much less common: their frequency ranged from 0.41% to 13.11%, depending on the type and group of lesions; The lowest frequency was characteristic of posterior (0.41-2.05%) and lateral/medial dislocations (1.64-3.69%), while rotational dislocations occurred somewhat more frequently, up to 13.11% in bilateral cases. 4. Effusion in the joint was most often observed in anterior and partial anterior dislocations. At the same time, in most cases, the level of effusion was minimal or small, and pronounced effusion was recorded only in isolated cases (up to 0.4%). 5. The absence of effusion in the presence of disc dislocation was frequent, especially with lateral, posterior, and rotational displacements. Specifically, in the unilateral group, effusion was absent in 75-88% of cases with these dislocation types, and similar proportions were observed in the bilateral group (absence in 55-80% of cases), confirming that effusion is not a typical finding outside of anterior and partial anterior displacements. 6. Bilateral lesions were associated with a higher incidence of both dislocations and effusion compared to unilateral lesions. For instance, anterior and partial anterior dislocations were detected in up to 17.2% and 15.6% of bilateral cases, respectively, versus 9.8% and 10.7% in unilateral cases. The rate of minimal or small effusion in bilateral dislocations was also up to twice as high as in unilateral cases (e.g., minimal effusion with anterior displacement: 5.3% in bilateral vs. 2.9% in unilateral joints). 7. The study results support a predominantly mechanical etiology for disc dislocation in this TMD cohort: the clear predominance of anterior dislocations, the high frequency of disc displacement without significant effusion (i.e., without clear MRI signs of acute inflammation), and the limited occurrence of moderate or pronounced effusion (0.4% of cases), all indicate that inflammatory processes play a minimal role in the majority of cases examined.

## Figures and Tables

**Table 1:** The frequency of TMJ disk dislocation.

Dislocation/trait	Right TMJ n (%)	Effusion(right): n (%)		LeftTMJ n (%)	Effusion(left): n (%)
Patients with unilateral dislocation (Group 1) 244 joints					
anterior disk displacement	24 (9.84%)	0: 12 (4.9%),1: 7 (2.9%),2: 4 (1.6%),3: 1 (0.4%),4: 0,5: 0.		21 (8.61%)	0: 9 (3.7%),1: 7 (2.9%),2: 3 (1.2%),3: 2 (0.8%),4: 0,5: 0.
partial anterior disk displacement (lat/med)	21 (8.61%)	0: 11 (4.5%),1: 6 (2.5%),2: 3 (1.2%),3: 1 (0.4%),4: 0,5: 0.		26 (10.66%)	0: 14 (5.7%),1: 7 (2.9%),2: 4 (1.6%),3: 1 (0.4%),4: 0,5: 0.
rotational anterolateral/anteromedial	15 (6.15%)	0: 8 (3.3%),1: 4 (1.6%),2: 2 (0.8%),3: 1 (0.4%),4: 0,5: 0.		16 (6.56%)	0: 8 (3.3%),1: 4 (1.6%),2: 2 (0.8%),3: 2 (0.8%),4: 0,5: 0.
lateral/medial disk displacement	4(1.64%)	0: 3 (1.2%),1: 1 (0.4%),2: 0,3: 0,4: 0,5: 0.		5(2.05%)	0: 3 (1.2%),1: 2 (0.8%),2: 0,3: 0,4: 0,5: 0.
posterior disk displacement	2(0.82%)	0: 2 (0.8%),1: 0,2: 0,3: 0,4: 0,5: 0.		3(1.23%)	0: 2 (0.8%),1: 1 (0.4%),2: 0,3: 0,4: 0,5: 0.
normal disc position (no displacement)	56 (22.95%)	0: 54 (22.1%),1: 2 (0.8%),2: 0,3: 0,4: 0,5: 0.		51 (20.90%)	0: 50 (20.5%),1: 1 (0.4%),2: 0,3: 0,4: 0,5: 0.
Patients with bilateral dislocation (Group 2) 244 joints					
Dislocation/trait	Right TMJ n (%)		Effusion(right): n (%)	LeftTMJ n (%)	Effusion(left): n (%)
anterior disk displacement	42 (17.21%)		0: 19 (7.8%),1: 13 (5.3%),2: 6 (2.5%),3: 3 (1.2%),4: 1 (0.4%),5: 0 (0.0%)	41 (16.80%)	0: 17 (7.0%),1: 13 (5.3%),2: 7 (2.9%),3: 3 (1.2%),4: 1 (0.4%),5: 0 (0.0%)
partial anterior disk displacement (lat/med)	37 (15.16%)		0: 15 (6.1%),1: 12 (4.9%),2: 7 (2.9%),3: 2 (0.8%),4: 1 (0.4%),5: 0 (0.0%)	38 (15.57%)	0: 16 (6.6%),1: 12 (4.9%),2: 7 (2.9%),3: 2 (0.8%),4: 1 (0.4%),5: 0 (0.0%)
rotational anterolateral/anteromedial	31 (12.70%)		0: 13 (5.3%),1: 11 (4.5%),2: 5 (2.0%),3: 2 (0.8%),4: 0 (0.0%),5: 0 (0.0%)	32 (13.11%)	0: 13 (5.3%),1: 11 (4.5%),2: 5 (2.0%),3: 2 (0.8%),4: 1 (0.4%),5: 0 (0.0%)
lateral/medial disk displacement	9(3.69%)		0: 5 (2.0%),1: 3 (1.2%),2: 1 (0.4%),3: 0 (0.0%),4: 0 (0.0%),5: 0 (0.0%)	8(3.28%)	0: 5 (2.0%),1: 2 (0.8%),2: 1 (0.4%),3: 0 (0.0%),4: 0 (0.0%),5: 0 (0.0%)
posterior disk displacement	1(0.41%)		0: 1 (0.4%),1: 0 (0.0%),2: 0 (0.0%),3: 0 (0.0%),4: 0 (0.0%),5: 0 (0.0%)	5 (2.05%)	0: 4 (1.6%),1: 1 (0.4%),2: 0 (0.0%),3: 0 (0.0%),4: 0 (0.0%),5: 0 (0.0%)

Notes:0 — none1 — minimum2 — a small amount3 — moderate4 — increased number5 – turns

## Data Availability

The datasets used and/or analyzed during the current study are available from the corresponding author.
